# Synthesis and Thermo-Mechanical Study of Epoxy Resin-Based Composites with Waste Fibers of Hemp as an Eco-Friendly Filler

**DOI:** 10.3390/polym13040503

**Published:** 2021-02-07

**Authors:** Mateusz Gargol, Tomasz Klepka, Łukasz Klapiszewski, Beata Podkościelna

**Affiliations:** 1 Department of Polymer Chemistry, Faculty of Chemistry, Institute of Chemical Science, Maria Curie-Skłodowska University, M. Curie-Skłodowska Sq. 3, PL-20031 Lublin, Poland; mateusz.gargol@poczta.umcs.lublin.pl; 2 Department of Technology and Polymer Processing, Faculty of Mechanical Engineering, Lublin University of Technology, Nadbystrzycka 36, PL-20618 Lublin, Poland; t.klepka@pollub.pl; 3 Faculty of Chemical Technology, Institute of Chemical Technology and Engineering, Berdychowo 4, Poznan University of Technology, PL-60965 Poznan, Poland; lukasz.klapiszewski@put.poznan.pl

**Keywords:** fibers of hemp, epoxy resin, epoxy resin composites, thermal and mechanical properties

## Abstract

The synthesis, thermal, and mechanical properties of epoxy resin composites incorporating waste fibers of hemp were studied. Five different systems with increasing quantity of the eco-filler were obtained. For the synthesis of polymeric materials, the commercial epoxy resins Epidian^®^ 5 and triethylenetetramine (TETA) were applied as crosslinking agents. The composites were obtained based on the polyaddition reaction of an amine group with an epoxide ring. ATR/FT-IR (Attenuated Total Reflection-Fourier Transform Infrared) analysis was used to confirm the chemical structure of the composites and the course of curing processes. Moreover, the influence of the eco-friendly components on the mechanical properties was determined, while thermal properties of the materials were investigated by thermogravimetry analysis (TGA) and differential scanning calorimetry (DSC). Dynamic mechanical studies (DMA) and Shore hardness tests of the obtained polymers were also carried out. The DSC curves and DMA analysis revealed that all materials were characterized by a similar glass transition range. Furthermore, the DMA and hardness measurements of the composites demonstrated an increasing elasticity with the increase in the amount of eco-filler present in the compositions.

## 1. Introduction

Nowadays, people possess greater awareness and approval of biodegradable, environmentally friendly materials, and recyclable products. Moreover, concerns about the environment protection and exploitation of nonrenewable resources during goods manufacturing are legislatively relevant issues in many countries. Therefore, natural materials should be used in manufacturing the products of everyday use whenever possible [1,2]. 

The aforementioned natural material products are mainly natural fiber-reinforced composites (NFRCs). Natural fibers in these materials provide several benefits as they are abundant, recyclable, and non-toxic with regard to soil ecology, people, and animals [3,4,5,6]. Natural fibers used as reinforcements are low cost compared with carbon fibers or glass fibers. Owing to this fact, natural fibers-reinforced composites can be affordably priced; thus, they are genuinely competitive components in many industrial branch processes [6,7,8]. Natural fibers can be classified based on their origin as mineral fibers, animal fibers, or plant fibers. Plant fibers are built from cellulose, hemicelluloses, and lignin. Natural fibers are used like light-weight fillers that significantly reduce the weight of materials (their densities are from 1.1 to 1.6 g/cm^3^) [9,10,11]. 

In designing the natural fibers of reinforced composites, common agricultural residues and biomass are exploited [12,13,14,15]. For reinforcement of the polymeric matrices (elastomers, thermoplastic, or thermosetting) rice husk, bamboo, wood, flax, hemp, cotton, pineapple leaves, and many others are utilized [16,17,18,19,20]. The thermo-mechanical parameters of the obtained composite are influenced by many factors, among others, fiber orientation and fiber length, chemical composition of the originating plant, fiber weight % or volume fraction, and fiber architecture. These parameters affect tensile and flexural strength, glass transition temperature, and thermal resistance [21,22,23]. Lionetto et al. described the morphology of injection molded short basalt fibers reinforced polypropylene in view the fiber length distribution and orientation. The results obtained from micro-CT analysis indicating that most of the fibers are aligned in the injection direction [24].

Stănescu and Bolcu [25] carried out the synthesis of composite materials with natural reinforcements (fabrics of flax, cotton, hemp, cattail leaves, and wheat straw) while the matrix were hybrid mixtures of epoxy resin and resin dammar (in different proportions). They compared their mechanical and the damping properties. The analysis showed a decrease in the values of the tensile strength and the modulus of elasticity as the dammar volume proportion was increased in the composite. Moreover, scientists noticed an increase in the damping capacity, with a higher dammar quantity in the sample composition. There are many reports in the literature about the use of fibers derived from grass. Vijaykumar et al., for example, attempted to use the *Eulaliopsis binata* grass as reinforcement for the polypropylene composites [26]. Atmakuri et al. characterized the mechanical and wettability properties of the reinforced natural fiber epoxy resin composites. Researchers fabricated hybrid composites based on the epoxy resin and fibers: Hemp, flax, banana, or pineapple. For comparison, the single-fiber composites and double-fiber composites of varying proportions of the fiber were synthesized. It was found that hybrid composites showed improved mechanical properties when compared to the pristine composites. As follows from the moisture analysis, all materials absorbed water and nature of their surface was hydrophilic. Based on the overall measurement results, the researchers proved that the hybrid composites were characterized by the improved properties compared to those of the pristine composites and that hemp and flax fibers could be a potential replacement for reinforcements in the composites [27].

The synthetic composites reinforced by natural fibers exhibit satisfactory durability compared to the glass or aluminum fibers, making them widely applicable e.g., in the automotive or aerospace industries or construction [28,29,30,31]. In some cases, the lower strength parameters are compensated by lower costs, weight, better damping, and greater environmental friendliness. 

Building construction is an important domain where natural fiber reinforced composites can be applied. These light-weight materials are suitable for construction of floor panels, tailgates trim, and dashboards [32,33,34,35]. The studies on incorporation of natural reinforcements in polymers, glass, and concrete to extend their functional properties and their application were also carried out. As a result, commercial construction materials (concrete, steel, wood) as well as daily products can be substituted by new composite materials [36,37,38,39,40]. 

In this article, synthesis and characterization of the crosslinked composites based on Epidian^®^ 5 and TETA with lower environmental impact by using waste fibers of hemp as the eco-filler are presented. The main objective of the study was to influence a biowaste from the hard part of stems (after oil production), on the physico-chemical properties of the obtained materials. The biowaste was mechanically fragmented before the use. The detailed thermal (TG, DSC), and thermo-mechanical (DMA) analyses, as well as the measurements of tensile and flexural strength, were made, showing how the addition of fibers effects on the resistance of the composites. Epoxy resins have many advantages e.g., they crosslink readily at room temperature and have excellent affinity for other materials. However, due to a high degree of crosslinking, they are poorly degradable materials in the environment. The use of the bio-based filler can significantly accelerate this process and make them more bio-friendly. 

## 2. Materials and Methods 

### 2.1. Materials

Epoxy resin: Epidian^®^ 5 (at 25 °C density: 1.17 g/cm^3^; viscosity: 20,000–30,000 mPas; epoxy number: 0.48–0.51 mol/100 g) was purchased from Ciech Sarzyna S.A. (Nowa Sarzyna, Poland) and was used as a monomer while triethylenetetramine (TETA) from Sigma-Aldrich (Steinheim am Albuch, Germany) acted as a crosslinking agent. Natural waste hemp fibers derived from production processes were used as an eco-filler. 

Hemp fibers (*Cannabis sativa*) came from the crops intended for oil production. The waste, in the form of a stem after drying, was subjected to mechanical fragmentation, the outcome of which were 8–20 mm long fibers, which were treated as a resin eco-filler. Fragmentation of the fibers is of practical importance during preparation of a composition in mold, and it the better mixing of the resin with the fibers and a more even distribution of the fibers in the resulting composite.

### 2.2. Methods

The fragments of the solid composites were studied using a Morphologi G3 optical microscope (Malvern, Great Britain).

Attenuated total reflection-Fourier transform infrared (ATR/FT-IR) spectra were recorded on a Bruker FT-IR spectrophotometer TENSOR 27 (Bruker GmbH, Mannheim, Germany), using powdered samples. Spectra were recorded from 4000 to 600 cm^−1^ with a resolution of 4 cm^−1^ and 32 scans. 

Thermal stability (TGA/DTG) was performed with a Netzsch STA 449 F1 Jupiter thermal analyzer (Netzsch, Selb, Germany) under the following operational conditions: the heating rate of 10 °C/min, dynamic atmosphere of helium (flow 20 cm^3^/min), temperature range of 25–600 °C, sample mass ~15 mg, and sensor thermocouple type S TG-DSC. All TGA/DTG measurements were taken in Al_2_O_3_ crucibles. As a reference, an empty Al_2_O_3_ crucible was used. 

Differential scanning calorimetry (DSC) curves were obtained on a DSC Netzsch 204 calorimeter (Netzsch, Günzbung, Germany). The samples (~15 mg) were placed in the aluminum pans with pierced lids. An empty crucible was used as a reference. Dynamic scans were obtained at a heating rate of 10 °C/min in the temperature range from 25 °C to 550 °C, within a nitrogen atmosphere (flow rate: 20 cm^3^/min).

Dynamic mechanical analysis (DMA) was performed using DMA Q800 Analyzer TA Instruments (New Castle, DE, USA). Thermo-mechanical properties of the cured materials were determined from the storage modulus, loss modulus, and damping factor (tan δ_max_) versus temperature. Measurements for all samples were made in the scanning temperatures ranging from 0 to 190 °C, under natural air conditions, at a constant heating rate of 4 °C/min. The experiments were conducted using rectangular samples of the dimensions close to 3 ± 0.1 mm thick, 5 ± 0.2 mm wide, and 35 ± 0.1 mm long. Mechanical properties were determined by means of a Zwick/Roell testing machine (model Z010, Zwick GmbH & Co. KG, Ulm, Germany). The specimen dimensions were 80 × 10 × 3 (±0.2) mm. The measurements were made at room temperature with a crosshead speed of 50 mm/min. All data were subjected to the analysis of variance using the Origin 8.1 (OriginLab, Northampton, MA, USA) applications. The one-way analysis of variance (one-way ANOVA) was used to detect significant differences among the tested mechanical parameters (Young’s module, stress at break, and relative elongation at break) depending on the fibers content.

Hardness of samples was compared by means of the Shore Hardness Tester Affriee in D scale, (model ART.13/serial Y5493, Omag, Italy). Five measurements were made for each sample and the average hardness was calculated for all samples. 

### 2.3. Curing Procedure

**Figure 1:**
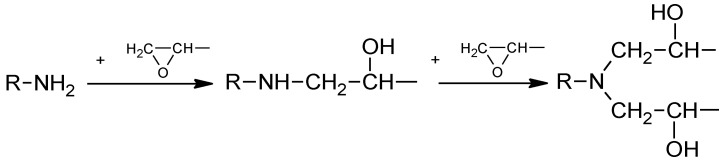
General scheme of the reaction of epoxy resin with amine.

Five epoxy based crosslinked materials containing: 0, 5, 10, 20, and 30 wt.% of the eco-filler were obtained based on the polyaddition reaction of an amine group with an epoxide ring (see Figure 1).

The ratio of the epoxy resin to the amine in each case was 10:1, (wt.%). The amount of amine for the epoxy number was determined previously in Ref. [38]. The experimental parameters of the syntheses are summarized in Table 1. 

**Figure 2:**
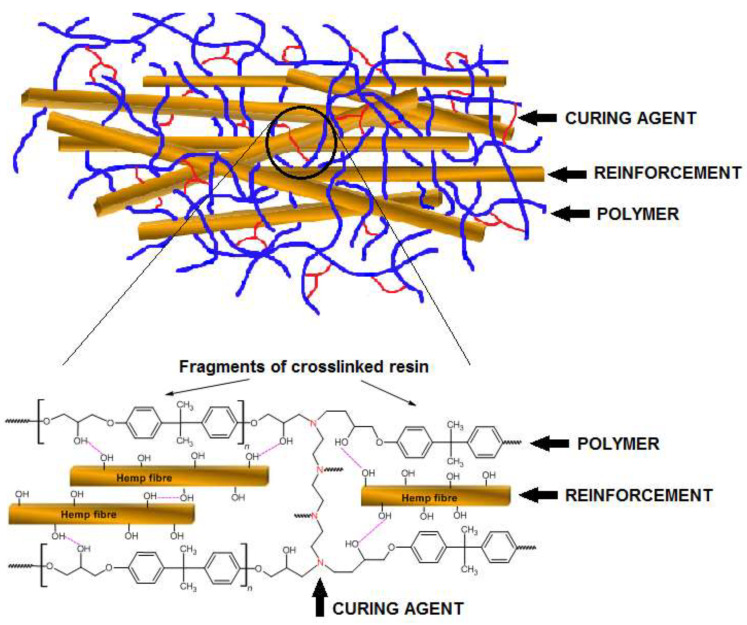
Simplified structure and interactions between components in the polymer composite with eco-filler.

Epidian^®^ 5 was weighed in suitable polyethylene containers. A specific amount of crosslinking agent was then added to each container and the content was thoroughly mixed. Next, the calculated amounts of eco-filler were added in small portions and the whole was thoroughly mixed. Finally, the contents were poured into similar rectangular glass molds to obtain identical samples. The fibers were added in the form of 8–20 mm fragments. The crosslinking process was conducted at room temperature for 10 h. The simplified structures of polymeric composites are visualized in Figure 2. The hemp fibers are made mainly of cellulose but also of lignin and hemicellulose. There are numerous hydroxyl groups in the cellulose that can affect the -OH groups present in the polymer chain, as proposed in Figure 2. These impacts can enhance the interactions of the fibers with the chains, contributing to their better adhesion to the resin. 

## 3. Results 

### 3.1. Optical Microscope Characterization

**Figure 3:**
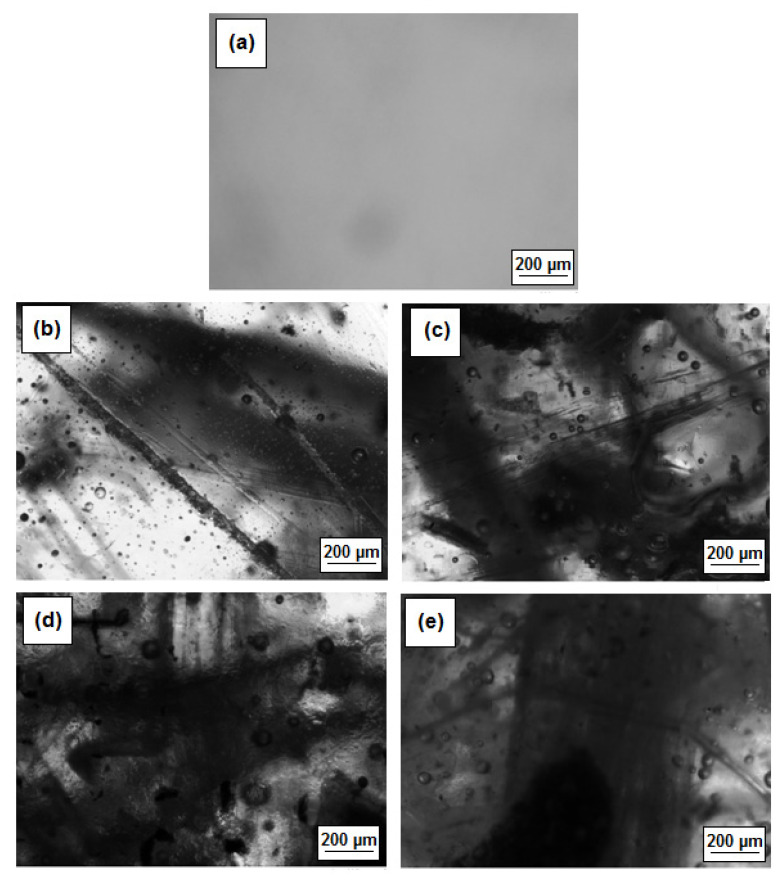
Microphotographs of the composites (**a**) sample without eco-filler; (**b**) sample with 5 wt.% of eco-filler; (**c**) sample with 10 wt.% of eco-filler; (**d**) sample with 20 wt.% of eco-filler; and (**e**) sample with 30 wt.% of eco-filler. Magnification 5×.

The photos of the obtained composites are presented in Figure 3. As one can see when the number of fibers increases, the composites become less transparent, but the fibers are clearly visible (even 30 wt.%) and embedded inside the material. The arrangement of fibers in the composite is random. The sample of pristine resin is transparent with a slight light-yellow shade. 

### 3.2. ATR/FT-IR Analysis

**Figure 4:**
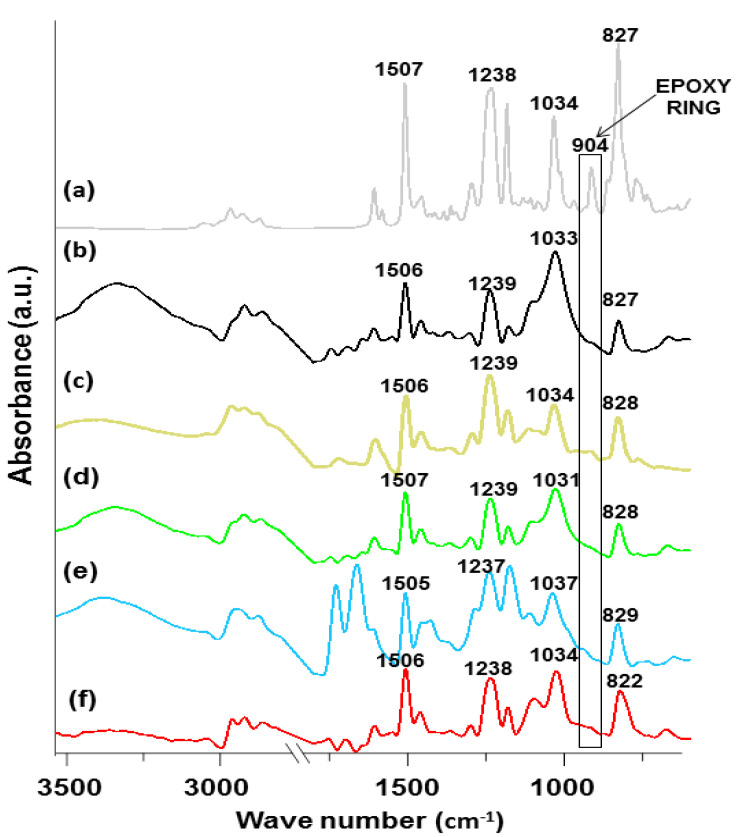
ATR/FT-IR spectra of the obtained materials: (**a**) Non-crosslinked epoxy resin; (**b**) sample without eco-filler; (**c**) sample with 30 wt.% of eco-filler; (**d**) sample with 20 wt.% of eco-filler; (**e**) sample with 10 wt.% of eco-filler and (**f**) sample with 5 wt.% of eco-filler.

Characterization of chemical structure by the spectroscopic analysis ATR/FT-IR (Attenuated Total Reflection-Fourier Transform Infrared spectroscopy) was made for all obtained materials. Figure 4 shows the spectra of the samples and Table 2 presents the wavelength values with the attributed intramolecular vibrations.

Epoxy resin and TETA are the fundamental components of the analyzed samples. Thus, characteristic bands of these reactants are pointed out on the spectra. In the 3342–3315 cm^−1^ range, wide bands derived from the stretching vibrations of the residual -OH groups or the adsorbed water are found. In the case of composites, it can be observed that the signal for the vibrations of -OH groups in the range 3415–3328 cm^−1^ is visible. This is due to the presence of numerous hydroxyl groups in the structure of cellulose (the main component of natural fibers). Additionally, after curing of the epoxy resin chains, -OH groups also exist (formed after opening the epoxy ring). Moreover, this signal can be more intensive due to the presence of amine (TETA) and strong tendency towards water absorption of these molecules. 

Characteristic bands of methyl and methylene groups are visible as two signals: The first peak from 2962 to 2925 cm^−1^ and the second in the range of 2922–2868 cm^−1^. These peaks correspond to the symmetrical and asymmetric stretching vibrations of both types of groups. There is a doublet of bands: 1605 and 1506 cm^−1^ corresponding to the symmetrical and asymmetric stretching vibrations of aromatic rings. Additionally, in the range 828–823 cm^−1^, the signal of deformation vibrations of Ar and Ar-H is notable. Its location indicates that carbon atoms number 1 and 4 are substituted in these rings. In the range from 1298 to 1284 cm^−1^, a band derived from the stretching vibrations of oxygen atoms connected with aromatic carbon atoms can be seen on the spectra. The bands at 1038–1026 cm^−1^ are associated with the stretching vibrations of hydroxyl groups in the neighborhood of primary carbon atoms, whereas the signals between 1112 and 1094 cm^−1^ come from the analogous stretching vibrations of hydroxyl groups connected with the secondary carbon atoms. Deformation vibrations of the mentioned two groups are visible in the range from 1458 to 1427 cm^−1^. The presence of the TETA-derived amine groups is confirmed by the valence band of stretching vibrations in the N-H bonds. This signal is observed from 730 to 648 cm^−1^. 

In the spectrum for non-crosslinked Epidian^®^ 5 (marked in Figure 4), a clear signal changing in intensity can be seen. This occurs around 914 cm^−1^ and can be assigned to the C-O deformation band in the epoxy group. After the crosslinking reaction, the lack of this signal is observed. Different courses of spectrum for the epoxy resin and the spectra for the crosslinked materials confirm the effectiveness of the crosslinking process [41].

### 3.3. Thermogravimetry Analysis

**Figure 5:**
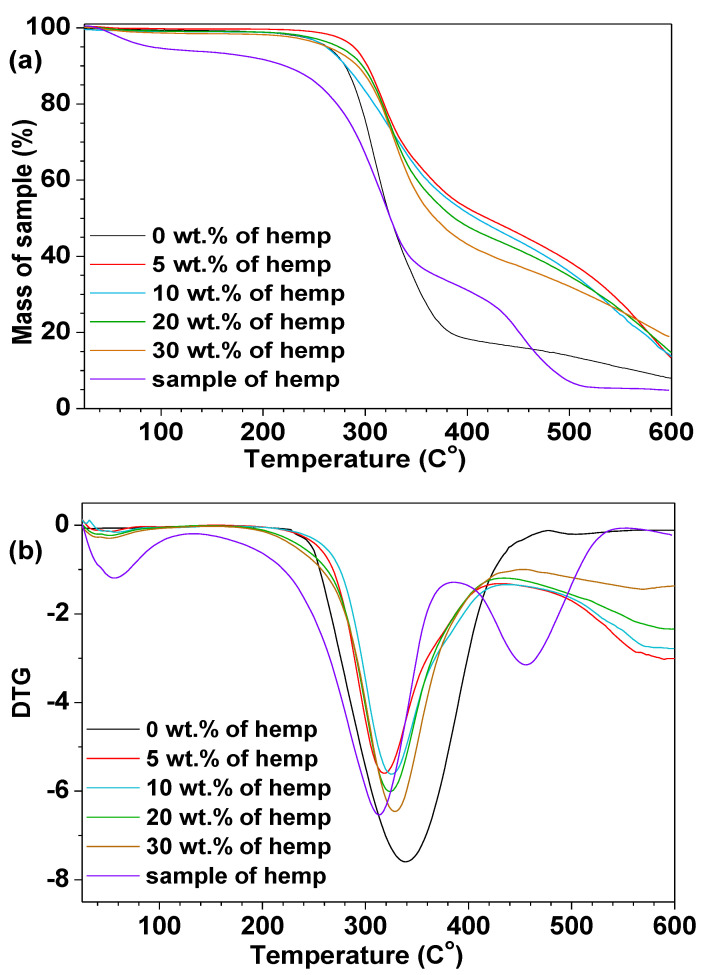
Thermal analysis: (**a**) TGA and (**b**) DTG curves of composites.

Thermal stabilities and degradation behavior of the polymeric composites were studied by means of thermogravimetry. The curves obtained from the TGA and DTG (differential) measurements (in helium) for all samples are presented in Figure 5. The mass loss factors—Initial Temperature of Decomposition (ITD)—corresponding to the temperature of 2% of mass loss, T_50%_ (temperature at 50% weight loss), maximum decomposition temperatures (T_1_ and T_max_) with the mass losses and the residual masses (RM) for each sample are listed in Table 3.

The thermal decomposition of hemp sample (bio-filler in the composites) proceeded in three stages. Three separate signals related to the degradation stages can be seen on the DTG curve. The first peak (T_1_) at 52.6 °C with the 1.58% mass loss was assigned to the volatilization of small amounts of unreacted monomers and water. The second peak at T_max_ 322.7 °C was related to the main degradation of the sample. The third decomposition stage can be also found on the DTG curve. This peak was observed at about 455 °C and was probably related to the degradation of lignin present in the hemp fibers. In the case of the sample without the eco-filler, the initial decomposition temperature is about 230 °C. The DTG curve for this sample contains one separate degradation step with the maximum of the mass loss (T_max_) at 337.9 °C and is related to the total degradation of the resin. The unreinforced sample had generally lower T_50%_ and ITD values compared to the modified composites. For the analyzed composites, the TGA and DTG curves had almost the same course up to a temperature of ca. 230 °C, all the composites were thermally stable (the range of ITD was from 213.1 to 247.7 °C). Further heating of the analyzed materials above T_max_ led to their complete thermal degradation. The highest thermal residue mass (RM) assessed at the final temperature was for the composite with 30 wt.% eco-filler (18.9%).

**Figure 6:**
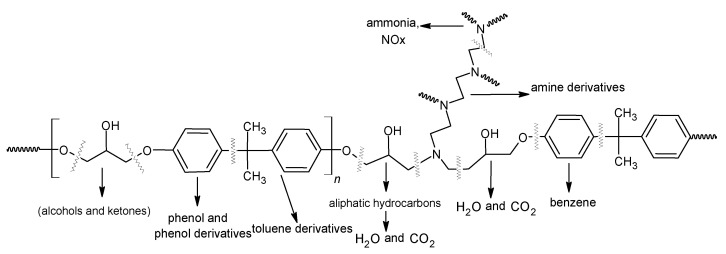
Proposal mechanisms of polymer fragmentation under heating.

In Figure 6, the proposed mechanism of polymer network fragmentation is presented. The suggested mechanism is based on our earlier research and literature data [42,43]. In the case of natural fillers, their decomposition leads to environmentally safe, small aliphatic hydrocarbons, alcohols, and then H_2_O and CO_2_. While crosslinking the epoxy resins, the main products of thermal decomposition, (apart from carbon dioxide and water) are phenol, phenol derivatives, benzene, toluene, amino derivatives, and nitrogen oxides.

### 3.4. DSC Analysis 

**Figure 7:**
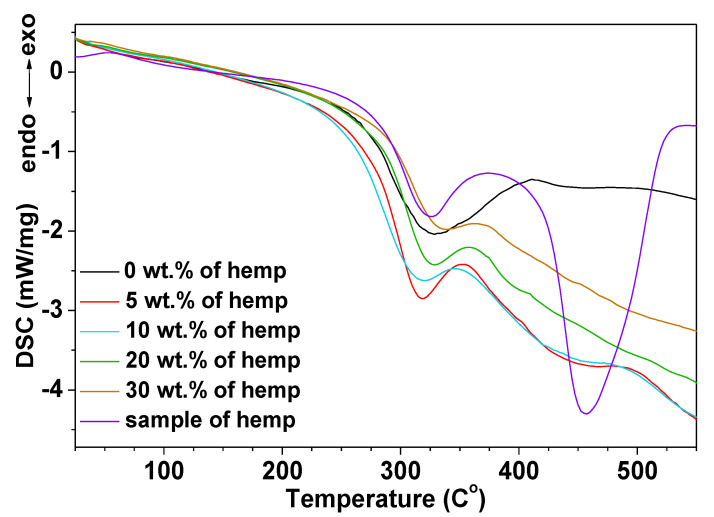
DSC curves of the obtained samples.

The thermal behavior of the obtained composites was also studied by means of DSC analysis. The DSC curves of these materials are presented in Figure 7. 

On the curve for the sample without eco-filler, one endothermic effect with the maximum at 332.4 °C could be seen. This endothermic effect was connected with the total thermal degradation of the sample. No exothermic effect (about 200 °C) associated with crosslinking was visible. The DSC curves of the composites with hemp fibers had a similar course. The maxima of the endothermic effect peaks occur in the temperature range from 318.4 to 339.1 °C. The addition of an eco-filler increases the decomposition temperature by about 7 °C in the case of 30 wt.% of eco-filler. The addition of natural filler affects positively the thermal resistance of the obtained materials. Additionally, pristine hemp fibers were also studied. On the curves, one can see two endothermic effects (325.7 and 457.4 °C) due to the thermal degradation. The resulting curve course (two maxima) is most likely associated with a large amount of aromatic lignin in the hardened parts of the hemp stem [42]. 

### 3.5. DMA Analysis 

**Figure 8:**
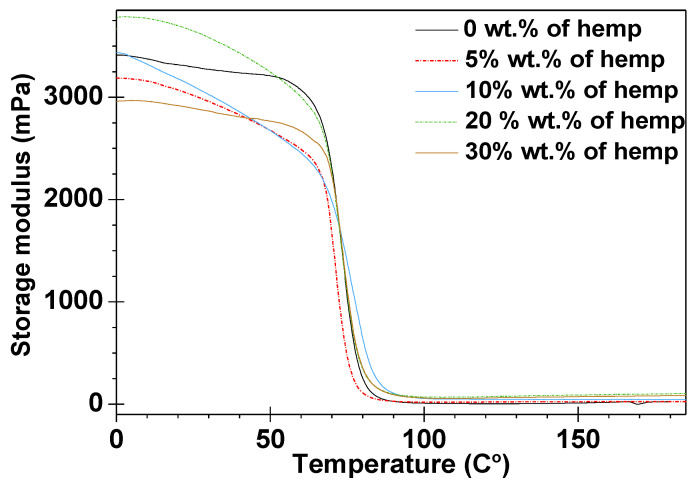
Storage modulus versus temperature for all compositions.

**Figure 9:**
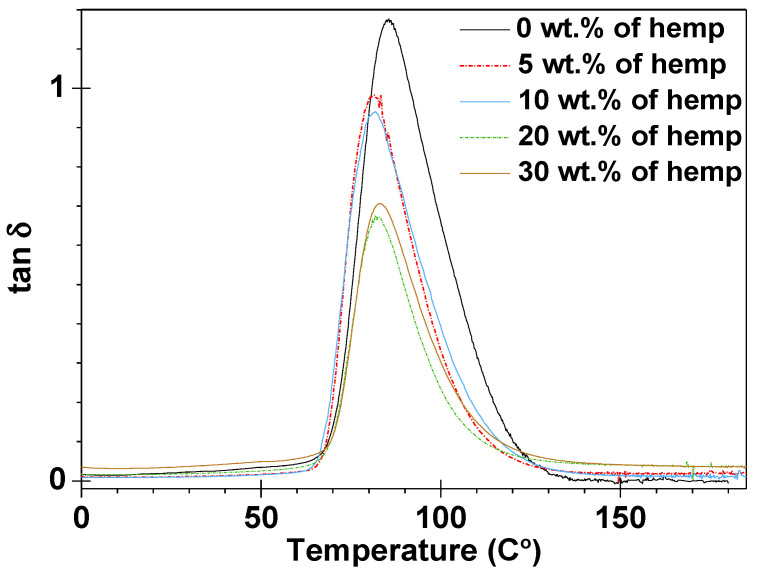
Tangent δ (tan δ) versus temperature for all compositions.

The results from the dynamic, mechanical, and thermal analyses of the obtained samples are presented in Figure 8 and Figure 9, as well as in Table 4.

The storage moduli (E’) in the function of temperature for the obtained five materials are depicted in Figure 8. Upon analyzing these values, major changes in the storage modulus are observed when the materials pass through the glassy to the rubber–elastic state. After the glass transition, the storage modulus no longer decreases, and plateaus are observed on all curves. In Figure 8, it is noticeable that the materials containing 5, 10, and 20 wt.% of eco-filler lose their storage moduli with the increase of temperature at higher rates than those of the composites with 30 wt.% of eco-filler. This indicates that for these three samples, the stiffness decreases more rapidly. Figure 9 also reveals the tan δ curves for four composites with different contents of eco-filler and for one cured epoxy resin. On examining the courses of the tan δ curves, it can be seen that the obtained samples exhibit symmetrical tan δ plots with only one maximum. In this paper, the position of the tan δ maximum was taken as the glass transition temperature T_g_ (associated with the process of segmental relaxation) [44]. In analyzing the tan delta values, it can be observed that the glass transition region spreads over a similar temperature range regardless of the composite type. 

The values of the damping factor (tan δ_max_) were in the range of 0.67–1.17 for the composites containing the epoxy resin as the matrix cured by TETA. The DMA plots show the highest value of tan δ for the sample without eco-filler. Measurements showed that the addition of filler reduces the damping capacity of energy during deformation [45]. The values of this parameter decrease with the increase in the percentage amount of eco-filler in the prepared compositions. As a result, the composite containing 5 wt.% of hemp had a similar damping factor value as the sample with 10% wt.% hemp. The same situation was found for the materials with the 20 wt.% and 30 wt.% eco-filler. 

Duc et al. [46] demonstrated that mechanical properties of polymer composites (storage modulus, loss factor) depend on many factors: Fibers type, polymer matrix type, and temperature of measurements. The researchers analyzed the mechanical properties of thermoset (epoxy) and thermoplastic (polypropylene PP and polylactic acid PLA) composites containing different fibers: Glass, carbon f, and flax fibers. Duc et al. observed that the addition of flax fibers to PP resulted in a decrease in the loss factor, indicating the damping properties of the matrix to be superior to those of the fibers. However, the damping increased when flax fibers was used instead of synthetic fibers in the epoxy composites. It can be inferred that the loss factor depends on the matrix and also on the fibers type. A similar trend was reported by Cicala et al. [47]. The scientists conducted DMA analysis of the epoxy composites with glass fibers, flax fibers, and hybrid reinforcement. As expected, the loss factor showed a different trend depending on the measurement temperature. Below the glass transition temperature, the sample reinforced by the flax fibers displayed a higher loss factor compared to that of the carbon fibers, while at the glass transition temperature, the opposite dependence was found. The composites with hybrid reinforcement displayed an intermediate behavior. This is an argument confirming the impact of temperature and type of fibers on the damping ability of polymer composites. 

**Figure 10:**
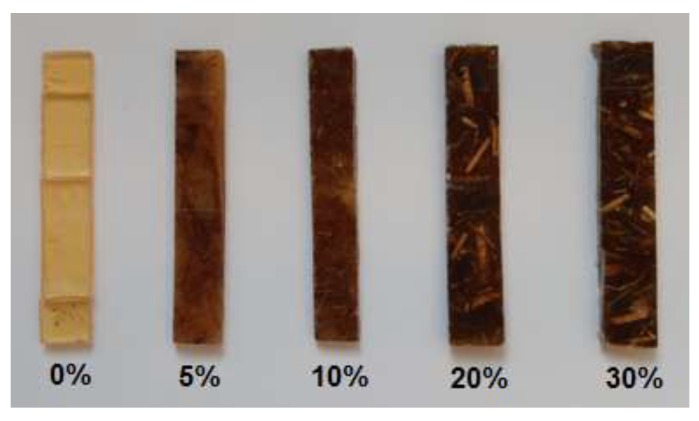
Photos of the studied samples after the DMA analysis.

The width of the peak tan δ in the middle of its height (FWHM, Full Width at Half Maximum) is a measure of the structure heterogeneity. The largest FWHM value was determined for the composite with 20 wt.% of eco-filler while the smallest value was characteristic of the sample without eco-filler. These transition regions could be a result of all samples showing a similar degree of structural heterogeneity. In Figure 10, the photos of the cut samples after the DMA analysis are presented. As one can see, only the sample without the hemp was cracked. In contrast, the samples with the addition of fibers retain their shape despite exposure to low-pressure forces during the analysis.

### 3.6. Tensile Test 

The tensile test consisted of the slow stretching of a sample (3 mm × 10 mm × 80 mm) with the addition of hemp fibers at a given constant speed in a uniaxial stretching system, using a Zwick/Roel Z010 universal testing machine. The test speed was 50 mm/min, and the tensile module speed was 5 mm/min. The Young’s modulus, yield stress, and elongation at break were determined. The crack resistance was designated during the three-point bending determining the modulus of flexural strength, the conventional yield strength, and bending deformation. The bending strength represents the largest stress generated in the material at the time of fracture. The tests were carried out on a Zwick Z010 testing machine with the bending test holders, the support spacing was 60 mm. The test speed and flexural module speed were 50 mm/min and 5 mm/min, respectively.

**Figure 11:**
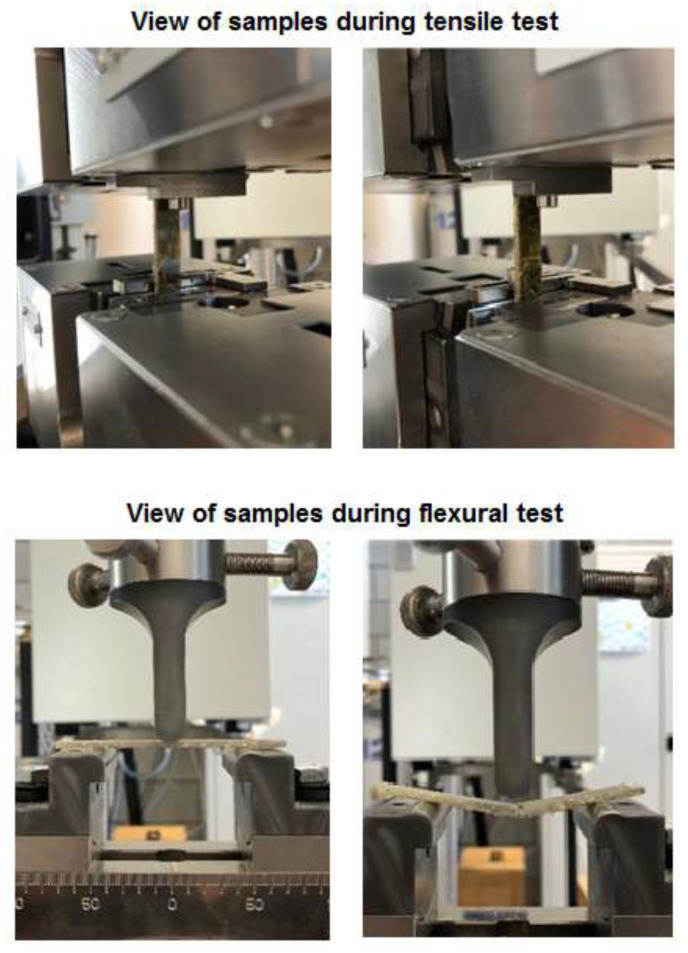
Exemplary samples during the mechanical measurements.

The results of tensile and flexural strength are presented in Table 5. Additionally, the exemplary samples during the tensile and flexural measurements are shown in Figure 11. As expected, the results of tests showed that the addition of hemp fibers to all materials causes a decrease in the value of the elastic modulus (Young’s modulus), which is the effect of a decrease in the tensile stress from 2790 to 1500 MPa. For the composites with the addition of 30% fibers, a significant decrease in the tensile strength is visible. This amount of eco-filler is too large; hence, the positioning of the fibers is incidental. Furthermore, this material may show too low resistance to external forces. 

The analysis of variance showed that only the Young modulus changes obtained at different percentage of fiber content were statistically significant, which was confirmed using the one-way analysis of variance (ANOVA) for the significance level *p* < 0.05. However, the changes in stress and elongation at break turned out to be statistically insignificant.

The mechanical strength was also dependent on the fiber distribution in the composite. Nevertheless, these changes are more evident during the tensile test. A large number of fibers (30%) reduces significantly the flexural strength from 102 (0 wt.%) to 25 MPa. However, due to the fact that the fibers were of considerable length (8–20 mm), while the samples cracked when subjected to the bending force, this did not break the continuity of both halves, which one can see in Figure 11 (view of the samples during the flexural tests). 

The addition of natural fibers (of various lengths), unlike glass, carbon, or basalt fibers, can have a smaller mechanical strength of the obtained composites [48,49] particularly in the case of highly crosslinked materials. However, as shown in Figure 11, the use of fibers indicates that these materials subjected to an external force maintain residual connections between the elements when cracking. This may be of practical use e.g., as elements of car bumpers. 

As described by Sathishkumar et al. [50] in the case of natural fibers, their influence on the mechanical properties of composites also depends on the chemical structure of the plant being used. Cellulose fibrils act as reinforcement in the plants and their amount determines the mechanical performance of the fibers used as a filler. In the case of major participation of lignin, present in the woody parts of the stem, the reductions of the mechanical properties are prominent, due to the interfacial effect between the fibers and the matrices. A similar behavior was observed in our previous studies using kraft lignin as an eco-filler in crosslinked composite materials [51].

### 3.7. Hardness Measurements 

**Figure 12:**
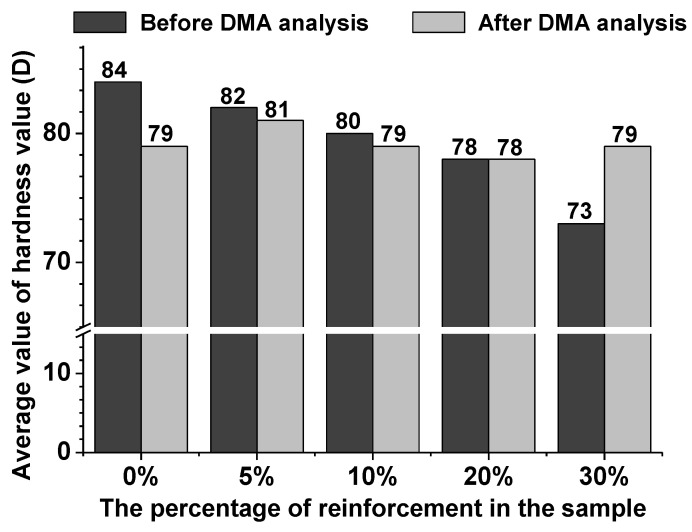
Hardness diagram of the obtained composites.

Measurements of hardness consisted in vertical immersion of the indenter into the composite surface. The numerical values of these parameters are expressed in the D scale in Figure 12 and Table 6.

Hardness of the composites before the DMA analysis was in the range of 73–84 units. The largest hardness was for the sample without eco-filler, while the lowest value was assigned to the sample with 30 wt.% of eco-filler. These results show the increasing ductility of the polymeric composites based on the epoxy resin content and the use of waste hemp fibers as the natural eco-filler. After the DMA analysis the following relationship was observed: The hardness of the composites with 5, 10, and 20 wt.% was almost without change whereas that for the materials with 30 wt.% of hemp was improved. This may be due to the fact that such a large amount of the eco-filler is a spatial problem and hinders the crosslinking process. The range of this parameter for the materials after DMA was 78–81 units in the D scale.

## 4. Conclusions

The objective of this article was the application of waste fibers of hemp as the eco-filler for the synthesis of more eco-friendly and thermal resistant polymeric composites based on the epoxy resin. The composites with different amounts of hemp fibers: 0, 5, 10, 20, and 30 wt.% were obtained, built upon the polyaddition reaction of an amine group with an epoxide ring. The TGA analysis demonstrated that for the analyzed composites, the TGA and DTG curves had almost the same trend. Up to a temperature of ca. 230 °C, all the composites were thermally stable (the range of ITD was from 213.1, to 247.7 °C). The largest thermal residue mass (RM) assessed at the final temperature was for the composite with 30 wt.% eco-filler. The addition of an ecological filler in the form of fibers positively influences the thermal resistance of composites. The DSC curves for the obtained samples show a similar trajectory and one endothermic signal corresponds to the thermal degradation of the samples. Upon analyzing the tan delta values from the DMA analysis, it can be observed that the glass transition region spreads over a similar temperature range regardless of the composite type. Moreover, the results from the DMA analysis and the values of hardness of the obtained materials show that the increasing amount of hemp reduces their hardness. With an increase of the eco-filler content, the material has a greater ability to vibration damping. As confirmed by the mechanical tests, hemp fibers can be used as eco-fillers in the polymer composites, but their quantity and form should be selected for their future application needs. As indicated by the research, it was also found that further measurements of both quantity and length of fibers should be made. Moreover, it could be beneficial to check not only curable plastics, but also thermoplastics as polymer matrices for fillers in the form of hemp fibers. These materials could be used for filling cavities or gaps in the polymer coatings and in the artificial, wood, or concrete surfaces. The addition of natural waste hemp fibers into the polymer materials can promote the sustainability of the plastic industry and increase the amount of environmentally friendly polymeric materials.

## Figures and Tables

**Table 1:** Experimental parameters of the syntheses.

Fibers Content (wt.%)	Epidian^®^ 5 (g)	TETA (g)	Hemp Fibers (g)
0	8.4	0.84	0.000
5	8.6	0.86	0.473
10	8.5	0.85	0.935
20	8.6	0.86	1.892
30	8.7	0.87	2.871

**Table 2:** Sample wavelength values with the attributed intramolecular vibrations.

	Fibers Content (wt.%)				
	0	5	10	20	30
Symbol of Vibration	Wave Number (cm^−1^)				
ν_–OH_	3315	3340	3342	3320	3334
ν-CH_3_, -CH_3_-	2963	2961	2948	2923	2925
	2922	2871	2875	2868	2869
ν-_C=O_	1727	1721	1724	1728	1723
ν-Ar	1605	1604	1605	1608	1604
	1506	1506	1505	1507	1506
δ-CH_3_, -CH_3_-	1455	1457	1458	1427	1452
ν-_C=O *aromat.*_	1297	1294	1295	1284	1298
ν-_CCO_	1239	1238	1237	1239	1239
ν-_C=O *alk.*_	1033	1030	1037	1031	1034
	1032	1033	1027	1038	1031
γ-_Ar, Ar-H_	828	827	823	829	828
γ-_N=H_	663	730	671	648	663

**Table 3:** TGA and DTG data of the samples.

TGA/DTG	Fibers Content (wt.%)					
	0	5	10	20	30	Only Hemp
T_1_ (°C)	58.1	52.4	57.5	52.0	50.3	52.6
T_1_ mass loss (%)	0.35	0.32	0.30	0.31	0.55	1.58
ITD (°C)	230.1	247.7	234.2	235.6	213.1	55.2
T_max_ (°C)	337.9	316.4	324.1	323.5	327.4	322.7
T_max_ mass loss (%)	59.3	18.2	25.9	24.6	29.2	51.2
T_50%_ (°C)	324.9	419.9	407.5	387.8	368.6	323.5
RM (%)	7.9	13.3	13.7	14.7	18.9	4.8

**Table 4:** Data of dynamic mechanical studies (DMA) measurements.

DMA	Fibers Content (wt.%)				
	0	5	10	20	30
T_g_ (°C)	84.1	81.4	81.6	82.3	82.5
damping factor/tan δ_max_	1.17	0.98	0.94	0.67	0.70
T_max Loss modulus_ (°C)	74.4	72.2	73.5	74.3	74.7
FWHD (°C)	25.7	22.1	25.5	21.1	22.9

**Table 5:** Tensile and flexural strength data of the samples.

	Fibers Content (wt.%)					
		0	5	10	20	30
	**Tensile Tests**					
Stress at break (MPa)	min	37.3	15	9.8	9.7	3.7
	avg	40.2	16.2	11.5	10.1	4.2
	max	41	17.5	12.3	11.9	4.7
Relative elongat. at break (%)	min	4.18	0.56	0.59	0.38	0.26
	avg	4.2	0.58	0.6	0.41	0.28
	max	4.23	0.6	0.61	0.43	0.3
Young’s modulus (MPa)	min	1465	2786	2417	2243	1498
	avg	1470	2790	2420	2250	1500
	max	1492	2802	2433	2257	1523
	**Flexural Tests**					
Stress at break (MPa)	min	99.5	42.2	39	38.9	24.8
	avg	102.1	43.3	39.5	40.3	25.2
	max	104.7	44	40.1	41	25.9
Relative elongat. at break (%)	min	2.2	1.07	0.97	0.94	0.87
	avg	2.4	1.2	1.1	1.1	0.9
	max	2.5	1.31	1.2	1.15	1.03
Young’s modulus (MPa)	min	4100	3343	3100	3243	2716
	avg	4110	3330	3120	3230	2720
	max	4123	3332	3129	3239	2730

**Table 6:** Values of hardness test of the synthesized samples.

Fibers Content (wt.%)	Hardness (°Sh) (D Scale)	
	Samples before DMA	Samples after DMA
0	84	79
5	82	81
10	80	79
20	78	78
30	73	79

## References

[1] Faruk O., Bledzki A.K., Fink H.P., Sain M. (2012). Biocomposites reinforced with natural fibers: 2000–2010. Prog. Polym. Sci..

[2] Girijappa Y.G.T., Rangappa S.M., Parameswaranpillai J., Siengchin S. (2019). Natural Fibers as Sustainable and Renewable Resource for Development of Eco-Friendly Composites: A Comprehensive Review. Front. Mater..

[3] Omrani E., Menezes P.L., Rohatgi P.K. (2016). State of the art on tribological behavior of polymer matrix composites reinforced with natural fibers in the green materials world. Int. J. Eng. Sci..

[4] Rachman M.M., Khan M.A. (2007). Surface treatment of coir (*Cocos nucifera*) fibers and its influence on the fibers physico-mechanical properties. Compos. Sci. Technol..

[5] Wambua P., Ivens J., Verpoest I. (2003). Natural fibers: Can they replace glass in fiber reinforced plastics?. Compos. Sci. Technol..

[6] Ishak M.R., Sapuan S.M., Leman Z., Rahman M.Z.A., Anwar U.M.K., Siregar J.P. (2013). Sugar palm (*Arenga pinnata*): Its fibers, polymers and composites. Carbohydr. Polym..

[7] Barari B., Omrani E., Dorri M.A., Menezes P.L., Pillai K.M., Rohatgi P.K. (2016). Mechanical, physical and tribological characterization of nano-cellulose fibers reinforced bio-epoxy composites: An attempt to fabricate and scale the ‘green’ composite. Carbohydr. Polym..

[8] Satyanarayana K.G., Arizaga G.G.C., Wypych F. (2009). Biodegradable composites based on lignocellulosic fibers—An overview. Prog. Polym. Sci..

[9] Mohammed L., Ansari M.N.M., Pua G., Jawaid M., Islam M.S. (2015). A review on natural fiber reinforced polymer composite and its applications. Int. J. Polym. Sci..

[10] Nagarajan V., Mohanty A.K., Misra M. (2013). Sustainable green composites: Value addition to agricultural residues and perennial grasses. ACS Sustain. Chem. Eng..

[11] Pappu A., Patil V., Jain S., Mahindrakar A., Haque R., Thakur V.K. (2015). Advances in industrial prospective of cellulosic macromolecules enriched banana biofiber resources: A review. Int. J. Biol. Macromol..

[12] Mittal V., Saini R., Sinha S. (2016). Natural fiber-mediated epoxy composites—A review. Compos. B Eng..

[13] Reddy N., Yang Y. (2009). Properties and potential applications of natural cellulose fibers from the bark of cotton stalks. Bioresour. Technol..

[14] Huda S., Yang Y. (2009). Feather fiber reinforced light-weight composites with good acoustic properties. J. Polym. Environ..

[15] Senthilkumar K., Saba N., Chandrasekar M., Jawaid M., Rajini N., Alothman O.Y., Siengchin S. (2019). Evaluation of mechanical and free vibration properties of the pineapple leaf fiber reinforced polyester composites. Constr. Build Mater..

[16] Zou Y., Huda S., Yang Y. (2010). Lightweight composites from long wheat straw and polypropylene web. Bioresour. Technol..

[17] Thakur V.K., Thakur M.K. (2014). Processing and characterization of natural cellulose fibers/thermoset polymer composites. Carbohydr. Polym..

[18] Jawaid M., Khalil H.P.S.A., Hassan A., Dungani R., Hadiyane A. (2013). Effect of jute fire loading on the mechanical and thermal properties of oil palm-epoxy composites. Compos. B Eng..

[19] Chee S.S., Jawaid M., Sultan M.T.H., Alothman O.Y., Abdullah L.C. (2019). Thermomechanical and dynamic mechanical properties of bamboo/woven kenaf mat reinforced epoxy hybrid composites. Compos. B Eng..

[20] Saba N., Jawaid M., Alothman O.Y., Paridah M.T. (2016). A review on dynamic mechanical properties of natural fiber reinforced polymer composites. Constr. Build. Mater..

[21] Senthilkumar K., Saba N., Rajini N., Chandrasekar M., Jawaid M., Siengchin S., Alotman O.Y. (2018). Mechanical properties evaluation of sisal fiber reinforced polymer composites: A review. Constr. Build. Mater..

[22] Safri S.N.A., Sultan M.T.H., Jawaid M., Jayakrishna K. (2018). Impact behaviour of hybrid composites for structural applications: A review. Compos. B Eng..

[23] Bolcu D., Stănescu M.M. (2019). The Influence of Non-Uniformities on the Mechanical Behavior of Hemp-Reinforced Composite Materials with a Dammar Matrix. Materials.

[24] Lionetto F., Montagna F., Natali D., De Pascalis F., Nacucchi M., Caretto F., Maffezzoli A. (2021). Correlation between elastic properties and morphology in short fiber composites by X-ray computed micro-tomography. Compos. Part A.

[25] Stănescu M.M., Bolcu D. (2019). A Study of Some Mechanical Properties of a Category of Composites with a Hybrid Matrix and Natural Reinforcements. Polymers.

[26] Vijaykumar G., Manikandan I., Adithya K., Akshay Koushik C.V., Srinivas C.V., Yogesh S., Nagananda G.S., Venkatesha K., Reddy N. (2019). Biofibers and biocomposites from sabai grass: A unique renewable resource. Carbohydr. Polym..

[27] Atmakuri A., Palevicius A., Siddabathula M., Vilkauskas A., Janusan G. (2020). Analysis of Mechanical and Wettability Properties of Natural Fiber-Reinforced Epoxy Hybrid Composites. Polymers.

[28] Stamboulis A., Baillie C.A., Peijs T. (2001). Effects of environmental conditions on mechanical and physical properties of flex fibers. Compos. A Appl. Sci. Manuf..

[29] Saba X.N., Paridah M.T., Jawaid M. (2015). Mechanical properties of kenaf fiber reinforced polymer composite: A review. Constr. Build. Mater..

[30] Nayak S.K., Mohanty S., Samal S.K. (2009). Influence of short bamboo/glass fiber on the thermal, dynamic mechanical and rheological properties of polypropylene hybrid composites. Mater. Sci. Eng. A.

[31] Di Bella G., Fiore V., Galtieri G., Borsellino C., Valenza A. (2014). Effects of natural fibers reinforcement in lime plasters (kenaf and sisal vs. Polypropylene). Constr. Build. Mater..

[32] Puglia D., Biagiotti J., Kenny J.M. (2004). A review on natural fiber-based composites—Part II: Application of natural reinforcements in composite materials for automotive industry. J. Nat. Fibers.

[33] Koronis G., Silva A., Fontul M. (2013). Green composites: A review of adequate materials for automotive applications. Compos. B Eng..

[34] Graupner N., Herrmann A.S., Müssig J. (2009). Natural and man-made cellulose fiber-reinforced poly(lactic acid) (PLA) composites: An overview about mechanical characteristics and application areas. Compos. A Appl. Sci. Manuf..

[35] Holbery J., Houston D. (2006). Natural-fiber reinforced polymer composites in auto- motive applications. JOM.

[36] Zini E., Scandola M. (2011). Green composites: An overview. Polym. Compos..

[37] Jawaid M., Khalil H.P.S.A. (2011). Effect of layering pattern on the dynamic mechanical properties and thermal degradation of oil palm-jute fibers reinforced epoxy hybrid composite. BioResources.

[38] Juárez C., Guevara B., Valdez P., Durán-Herrera A. (2010). Mechanical properties of natural fibers reinforced sustainable masonry. Constr. Build. Mater..

[39] Shah D.U. (2013). Developing plant fiber composites for structural applications by optimising composite parameters: A critical review. J. Mater. Sci..

[40] Gargol M., Podkościelna B. (2019). The use of waste materials as fillers in polymer composites—Synthesis and thermal properties. Physicochem. Probl. Miner. Process..

[41] Silverstein R.M., Webster F.X., Kiemle D.J. (2005). Spectrometric Identification of Organic Compounds.

[42] Sobiesiak M., Podkościelna B., Sevastyanova O. (2017). Thermal degradation behavior of lignin-modified porous styrene-divinylbenzene and styrene-bisphenol A glycerolate diacrylate copolymer microspheres. J. Anal. Appl. Pyrolysis..

[43] Salasinska K., Barczewski M., Borucka M., Górny L.R., Kozikowski P., Celiński M., Gajek A. (2019). Thermal stability, fire and smoke behaviour of epoxy composites modified with plant waste fillers. Polymers.

[44] Jones D.S. (1999). Dynamic mechanical analysis of polymeric systems of pharmaceutical and biomedical significance. Int. J. Pharm..

[45] Pączkowski P., Puszka A., Gawdzik B. (2020). Green Composites Based on Unsaturated Polyester Resin from Recycled Poly(Ethylene Terephthalate) with Wood Flour as Filler—Synthesis, Characterization and Aging Effect. Polymers.

[46] Duc F., Bourban P.E., Plummer C.J.G., Månson J.A.E. (2014). Damping of thermoset and thermoplastic flax fibre composites. Compos. Part A Appl. Sci. Manuf..

[47] Cicala G., Pergolizzi E., Piscopo F., Carbone D., Recca G. (2018). Hybrid composites manufactured by resin infusion with a fully recyclable bioepoxy resin. Compos. Part B: Eng..

[48] Matykiewicz D. (2020). Hybrid Epoxy Composites with Both Powder and Fiber Filler: A Review of Mechanical and Thermomechanical Properties. Materials.

[49] Merighi S., Mazzocchetti L., Tiziana Benelli T., Giorgini L. (2020). Adenine as Epoxy Resin Hardener for Sustainable Composites Production with Recycled Carbon Fibers and Cellulosic Fibers. Polymers.

[50] Sathishkumar G.K., Ibrahim M., Akheel M.M., Rajkumar G., Gopinath B., Karpagam R., Karthik P., Charles M.M., Gautham G., Shankar G.G. (2020). Synthesis and Mechanical Properties of Natural Fiber Reinforced Epoxy/Polyester/Polypropylene Composites: A Review. J. Nat. Fibers.

[51] Podkościelna B., Wnuczek K., Goliszek M., Klepka T., Dziuba K. (2020). Flammability Tests and Investigations of Properties of Lignin-Containing Polymer Composites Based on Acrylates. Molecules.

